# Penetrating dural venous sinus injury: a scoping review of clinical presentation, operative management, and prognosis

**DOI:** 10.1007/s10143-026-04451-2

**Published:** 2026-09-03

**Authors:** Yaxel Levin-Carrion, Jayant Bhasin, Taylor Campbell, Ebrahim Faizullabhoy, Kevin Titkov, Carlos Coronado-Guzman, Arman Sawnhey, Sraavya Anne, Daniela A. Perez-Chadid, Drew Thibault, Alejandro Pando, Nemanja Novakovic, Hai Sun

**Affiliations:** 1 Department of Neurological Surgery, Rutgers New Jersey Medical School, 185 S Orange Ave, Newark, NJ 07103 USA; 2 Department of Neurological Surgery, Rutgers Robert Wood Johnson Medical School, New Brunswick, NJ USA; 3 Department of Neurological Surgery, Corewell Health William Beaumont University Hospital, Royal Oak, MI USA

**Keywords:** Superior sagittal sinus, Transverse sinus, Torcula, Penetrating brain injury, Gunshot wound, Nail gun

## Abstract

Penetrating injury to the dural venous sinuses (DVS) commonly occurs at the superior sagittal sinus (SSS), transverse sinus (TS), and torcular confluence. While rare, DVS penetrating injuries carry high risk of intracranial hemorrhage, air embolism, and venous infarction. The current literature pertaining to penetrating DVS injury consists primarily of isolated case reports and small series. This scoping review consolidates published cases to characterize mechanisms, management strategies, and clinical outcomes pertaining to penetrating DVS injury. A scoping literature search of PubMed, PubMed Central, Embase, and Scopus was performed through September 2025. English-language case reports, case series, and retrospective studies describing penetrating cranial trauma involving the SSS, TS, or torcula were included. Data were summarized descriptively. Twenty-five studies met inclusion criteria, encompassing low-velocity penetrating injuries, civilian firearm trauma, captive-bolt injuries, and wartime shrapnel. Low-velocity injuries were typically managed with wide exposure, circumferential sinus control, and either flow-preserving repair or selective SSS ligation, with favorable neurological outcomes. Firearm-related injuries demonstrated greater variability, ranging from excellent recovery after torcular reconstruction to delayed sinus thrombosis and fatal pulmonary air embolism following incomplete SSS sealing. Reconstruction was generally favored for posterior SSS, torcular, and dominant TS injuries, whereas ligation was better tolerated for anterior or mid-SSS lesions. Penetrating DVS injuries require early venous imaging, meticulous sinus control, and segment-specific decisions regarding repair versus ligation. Flow-preserving reconstruction is increasingly feasible and may optimize survival and neurological recovery.

**Supplementary Information:**

The online version contains supplementary material available at https://doi.org/10.1007/s10143-026-04451-2.

## Introduction

Injuries to the dural venous sinuses are amongst the most serious complications of cranial trauma, with mortality estimates exceeding 40% of all traumatic head injuries with sinus involvement [1]. Dural venous sinuses are endothelium-lined channels formed by the separation of meningeal and periosteal dural layers and are therefore valveless and lack muscular vessel walls. These features, along with their role in draining the majority of cerebral venous blood and cerebrospinal fluid, render them uniquely susceptible to massive hemorrhage following laceration. Furthermore, the superior sagittal sinus (SSS), transverse sinus (TS), and torcular confluence are amongst the largest and most superficial structures, placing them at substantial risk during penetrating trauma.

Although penetrating head trauma accounts for only a small percentage of all traumatic brain injuries, it carries extraordinary mortality, with only a third of patients surviving to reach a hospital [2]. Firearm-related injuries demonstrate particularly dire statistics: historical series report 92% overall mortality when including both immediate and in-hospital mortality [3]. Amongst those reaching definitive neurosurgical care, concurrent vascular injuries including dural venous sinus lacerations account for approximately 30% of deaths [4]. Clinical consequences of sinus injuries often extend beyond hemorrhage. Occlusion of the posterior SSS, torcula, and dominant TS can precipitate intracranial hypertension, venous congestion, and potentially catastrophic hemorrhagic venous infarction [5]. Critically, the posterior third of the SSS possesses limited collateral drainage; occlusion therefore results in bilateral venous infarcts of the parietal and frontal lobes [6]. Penetrating sinus injuries also carry risk for venous air embolism, especially in cases when the head is elevated above the heart [7].

Outcomes from penetrating dural sinus injuries have been historically poor. Early military surgeons often resorted to sinus ligation or packing when direct repair proved unfeasibly, accepting high rates of venous infarction and mortality as inevitable. Modern neurosurgical practice has since achieved a paradigm shift; microsurgical techniques and specialized instruments now permit precise hemostasis [8]. Synthetics grafts such as expanded polytetrafluoroethylene provide robust reconstruction conduits and hemostatic adjuncts including gelatin-thrombin matrix, fibrin glue, and oxidized cellulose have revolutionized bleeding control [9–11]. Furthermore, advanced imaging including computed tomography venography, magnetic resonance venography, and digital subtraction angiography now permit preoperative sinus mapping and postoperative surveillance for delayed thrombosis [12, 13].

Despite these advances, evidence regarding optimal management of penetrating dural venous sinus injuries remains scarce due to the rarity of these cases precluding randomized controlled trials. Critical questions persist regarding timing of anticoagulation, comparative efficacy of reconstructive materials, and long-term outcomes following different strategies. This scoping review consolidates available evidence from case reports and institutional series to identify operative strategies, imaging decision points, complications, and outcome predictors to guide contemporary management.

## Methods

### Protocol, eligibility criteria, and study selection

This review was conducted in accordance with PRISMA 2020 guidance. An a priori protocol specified objectives, eligibility criteria, outcomes, and analysis plans.

The review question was prespecified as: among patients of any age with gun projectile/gunshot head trauma, what are the mechanisms, imaging findings, operative strategies, complications, and clinical outcomes when the dural venous sinuses (SSS. TS, or torcula) are injured?

Primary clinical evidence was accepted in the form of case reports, small case series, and retrospective cohorts, as higher-level comparative data are not available for this rare injury pattern. Primary outcomes of interest were survival and neurological recovery, while secondary outcomes included hemorrhage control strategy (repair vs ligation), venous patency, need for decompression, anticoagulation practices in traumatic dural venous sinus thrombosis (DVST), and major complications.

Eligibility criteria included: (i) clinical report (case report/series/cohort) of penetrating cranial trauma with direct involvement of a dural venous sinus (SSS,TS, or torcula) or a penetrating trajectory that produced extrinsic sinus compression with venous outflow obstruction or post-traumatic DVST attributable to penetration; (ii) sufficient clinical detail to ascertain mechanism, sinus segment, operative or imaging management, and at least one clinical outcome; and (iii) English language. We excluded purely blunt trauma without sinus penetration or compression, non-sinus vascular injuries, animal experiments, editorial or commentary without primary data, and non-English publications.

Study selection was performed in two stages (title/abstract, then full text) by two independent reviewers. Discrepancies were resolved by consensus. Titles and abstracts were screened against the eligibility criteria, and full texts were retrieved when abstracts suggested possible inclusion or were ambiguous. Review articles and technique notes without primary cases were included as contextual evidence in the narrative but did not contribute primary patient counts.

Because this review synthesizes published data only, institutional review board approval and patient consent were waived.

### Data sources and search strategy

We searched PubMed, PubMed Central, Embase, and Scopus from database inception to September 9, 2025, limited to English-language records using strings focused on dural sinus anatomy and penetrating trauma. Full search strings and results are listed in Supplemental Table 1.

### Data extraction, risk of bias and synthesis

Data extraction was performed with a standardized form that captured study design; number of patients; age/sex when available; mechanism (e.g., gunshot, nail-gun, drywall screw, captive bolt, shrapnel); sinus segment (SSS anterior/middle/posterior, TS, torcula); lesion type (laceration, transfixion, transection, extrinsic compression, DVST); imaging modalities (CT, CTA, CTV, MRV, catheter angiography, Q-MRV); operative strategies) e.g., donut or double-concentric craniotomy, proximal/distal control, repair versus ligation, reconstruction materials such as fascia/muscle, sutured patches, Gore-Tex, PTFE interposition), need for decompression; perioperative anticoagulation or thrombolysis; complications (air embolism, bullet embolism); and outcomes (survival, neurological recovery, shunt dependence).

**Fig. 1:**
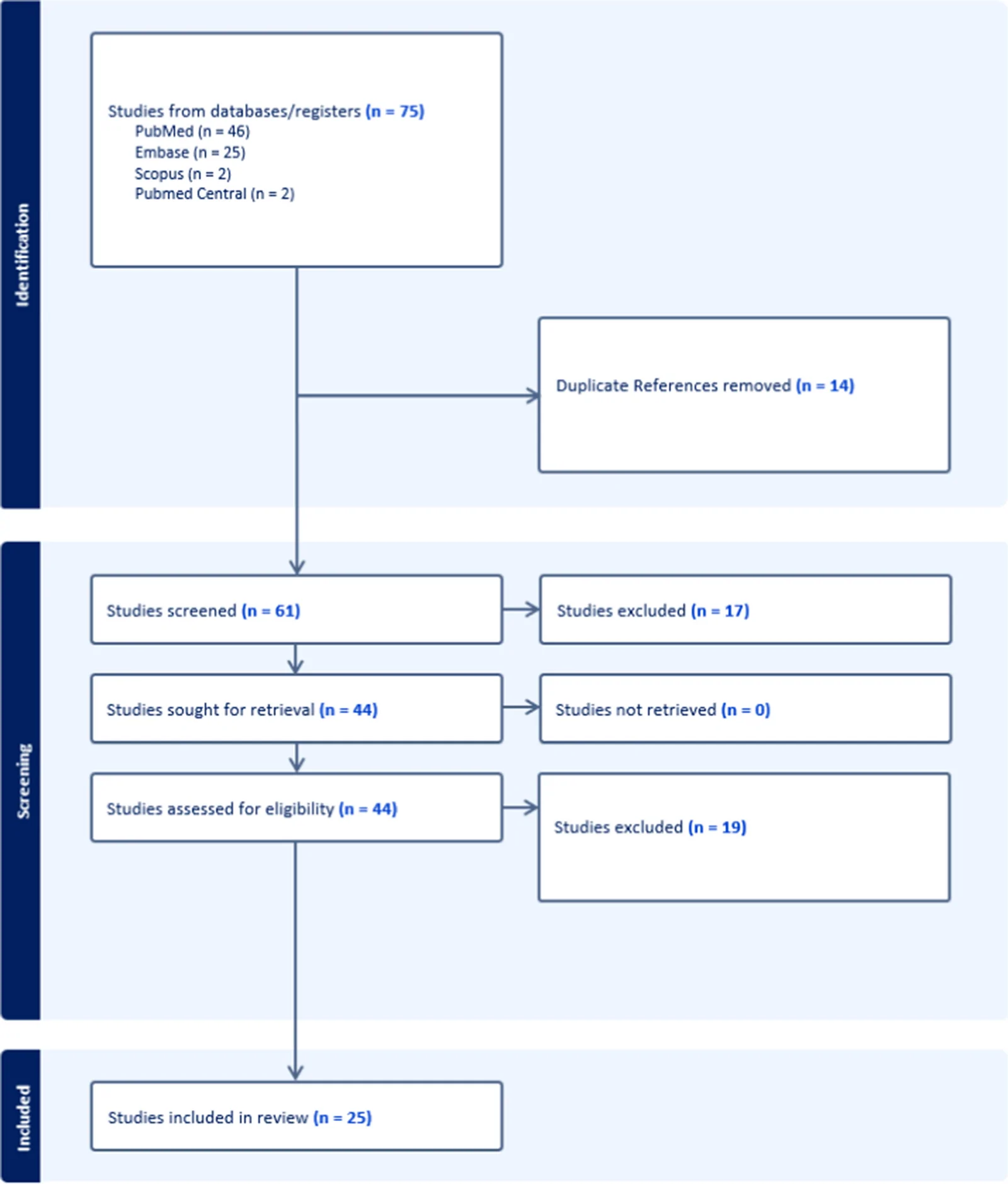
PRISMA flowchart of included studies

Risk of bias was assessed with the Joanna Briggs Institute (JBI) Critical Appraisal Checklist for Case Reports and the JBI Critical Appraisal Checklist for Cohort Studies. In total, 24 studies underwent appraisal, including 22 case reports and 2 cohort studies. Of the 22 case reports, 21 were considered appropriate for inclusion, while one study (Thoeny et al.) was excluded because several important elements were not adequately reported, particularly patient demographics, the clinical timeline, postintervention status, and adverse events. Among the case reports, the clearest and most consistently reported items were the presenting condition, diagnostic evaluation, treatment, and clinical lessons. Diagnostic testing was adequately described in all 22 reports, the intervention in 19, the postintervention condition in 17, and adverse or unexpected events in 18. The main weaknesses were incomplete demographic reporting and poor presentation of the sequence of clinical events. Only 1 of the 22 reports fully met the demographic domain, and only 8 clearly laid out the patient course in a structured timeline. The 2 cohort studies were generally stronger methodologically, with appropriate assessment of exposures and outcomes and reasonable statistical analysis, although 1 had unclear adjustment for confounding and both had some uncertainty regarding whether follow-up was long enough. Overall, the studies were of sufficient quality to allow qualitative synthesis, but the strength of the evidence remains limited by the case-based nature of the literature, incomplete reporting in several domains, and the selection and publication bias that are difficult to avoid in rare neurosurgical conditions. Because the data derive from single cases and small series, effect measures were not pooled; meta-analysis was not attempted. Instead, we performed a narrative synthesis with content analysis, organizing findings by mechanism (gunshot versus low-velocity penetrators) and by sinus segment (anterior/middle SSS versus posterior SSS/torcula/dominant TS), and highlighting recurrent operative tactics, imaging decision points, and complication patterns. Finally, we documented the study-selection flow to correspond with a PRISMA flow diagram (Fig. 1). Study characteristics are shown in Table 1.

**Table 1:** Demographics and Injury Patterns

Citation	Study type	Mechanism	Sinus involved	Segment	Lesion type	Age	Sex
Brune et al. [25]	Case report	Gunshot wound	SSS	Not specified (laceration)	Laceration; non-watertight closure	39	M
Birk et al. [22]	Case report	Gunshot wound (depressed fracture)	SSS	Posterior third	Extrinsic compression/outflow compromise	25	F
Pricola et al. [20]	Case report	Gunshot wound	Torcula (confluence)	Torcular region	Retained bullet; torcular dural defect; thrombosis	21	M
Haider et al. [21]	Case report	Gunshot wound (self-inflicted)	Torcula	Torcular region (extrinsic narrowing)	Extrinsic compression by depressed occipital fracture	27	M
Beaty et al. [23]	Case report	Gunshot wound	Transverse sinus	Right TS	Transection	23	M
Duke et al. [27]	Case report	Gunshot wound (BB)	Transverse sinus (initial location)	Right TS	Bullet embolism from TS to pulmonary artery	25	M
Duda et al. [32]	Case report	Gunshot wound	Not specified (venous entry suspected)	NA	Bullet fragment migration to right ventricle (acute)	“Middle-aged”	M
Sani et al. [14]	Case report	Nail-gun	SSS	Parietal	Transfixion/laceration	37	M
Fujiyama et al. [15]	Case report	Nail-gun	SSS	Anterior–mid	Transfixion/intrafalcine	25	M
Kow et al. [16]	Case report	Nail-gun (self-inflicted)	SSS	Anterior third	Transfixion + AV shunt	35	M
Guppy and Ochi [17]	Case report	Drywall screws (self-inflicted)	SSS	Not specified	Transfixion	30	M
Schlag et al. [18]	Case report	Captive bolt	SSS	Middle/posterior	Rupture with proximal/distal thrombosis	56	M
Okada et al. [19]	Case report	Nail-gun	Transverse sinus (edge)	TS (near)	Adjacent trajectory; sinus at risk	46	M
Abdallah et al. [9]	Case report (resource-limited)	Shrapnel (war)	SSS	Junction middle/posterior	Laceration with segmental loss	33	M
Arham and Zaragita [34]	Case report + literature review (context)	Nail-gun (index) + prior cases	SSS	Not specified	Penetration; hemostatic repair	3	M
Vakil and Singh [31]	Imaging review	Penetrating brain trauma (general)	DVS complications included	NA	Imaging evaluation; complications overview	NA	NA
Thoeny et al. [28]	Case report	Nail-gun	SSS	NA	Operative planning; air-embolism preparedness	28	M
Nussbaum et al. [26]	Technical note (SSS repair after nail-gun)	Nail-gun	SSS	Not specified	Penetration/laceration	45	M
Quiñones-Hinojosa et al. [29]	Case report + literature context	Penetrating trauma (GSW) with DVST	Transverse sinus	TS	Post-traumatic sinus thrombosis	48	M
Meyer et al. [37]	Prospective multicenter diagnostic cohort	Penetrating brain injury (mixed)	Cerebrovascular injury (arterial/venous)	NA	Imaging comparison (CTA vs DSA)		
Zafonte et al. [24]	Case series (3 patients)	GSW	SSS (± TS/straight)	Posterior SSS commonly	DVST (immediate/delayed/chronic)	18	F
Zafonte et al. [24]	Case series (3 patients)	GSW				16	F
Zafonte et al. [24]	Case series (3 patients)	GSW				27	F
Wang et al. [35]	Case report + review (nail-gun SSS)	Nail-gun (right parietal SSS)	SSS	Right parietal	Penetration; managed surgically	18	M
Suhendar et al. [33]	Case report	Low-velocity air gun	Right transverse sinus	Lateral transverse sinus	Penetrating sinus injury with sinus transaction/laceration and retained bullet		
Zhu et al. [36]	Case report	Self‑inflicted bolt‑gun injury	Superior sagittal sinus	Anterior third	Penetrating tract; SSS laceration; frontal bone fracture; intracranial hemorrhage		
Mansour et al. [30]	Retrospective cohort study	Civilian penetrating brain injury (mostly gunshot wounds)	Superior sagittal sinus most common; also transverse & sigmoid	Not specified	Occlusion (most common); impingement; transection		

## Results

A total of 75 non-duplicate records were identified through our comprehensive search. After title/abstract screening, 44 full-text articles were assessed for eligibility. Of these, 25 studies met inclusion criteria and were retained for qualitative synthesis. The most common reasons for exclusion were absence of dural venous sinus involvement, non-penetrating mechanisms, and lack of extractable patient-level data.

### Demographics and injury patterns

Across the 24 patient-level cases (Table 1), age data were available for 23, with a mean age of 28.6 years (SD 12.7; median 27). Most patients were male (20/24; 83.3%). The most common mechanisms of injury were gunshot/ballistic injuries (12/24; 50.0%; including one low-velocity air-gun injury), followed by nail-gun injuries (8/24; 33.3%). Drywall screw, shrapnel, captive-bolt, and bolt-gun injuries each accounted for 1 case each (4.2%)/The most affected sinus was the superior sagittal sinus (16/24; 66.7%), followed by the transverse sinus (6/24; 25.0%) and the torcula/confluence (2/24; 8.3%); while one case had unspecified venous entry. Among the 11 GSW cases, sinus involvement was more heterogeneous (SSS 5/11, 45.5%; transverse 5/11, 36.4%; torcula 2/11, 18.2%), whereas nail-gun injuries predominantly involved the SSS (⅞; 87.5%). Lesion descriptors were heterogeneous and not mutually exclusive: laceration/penetration/transection was reported in 9/24 cases (37.5%), thrombosis in 6/24 (25.0%), transfixion in 4/24 (16.7%), and embolic migration or retained foreign body in 4/24 (16.7%), and embolic migration or retained foreign body in 4/24 (16.7%).

### Imaging

Imaging was documented in 23/24 cases (Table 2). CT was the most common modality (16/23; 69.6%), followed by CTA (10/23; 43.5%), catheter angiography/DSA (7/23; 30.4%), CTV (5/23; 21.7%), and MRV (3/23; 13.0%); single cases used MRI and MRA. When summarized as the number of distinct radiographic modalities documented per case, two modalities were most common (12/23; 52.2%), followed by one modality (6/23; 26.1%), three modalities (4/23; 17.4%), and four modalities (1/23; 4.3%). No case documented five distinct imaging modalities.

**Table 2:** Imaging

Citation	Modality	Findings
Brune et al. [25]	Clinical course + autopsy	pulmonary air embolism
Birk et al. [22]	DSA showing; Q-MRV improvement post-op	compromised venous outflow
Pricola et al. [20]	CT/CTA; intraoperative findings	
Haider et al. [21]	CTV; serial ophthalmology exams	tapering near torcula
Beaty et al. [23]	CTA	
Duke et al. [27]	Head CT/CTA; serial chest X-rays; chest CT	
Duda et al. [32]	Serial CT; chest X-ray; chest CT showing fragment in right ventricle	
Sani et al. [14]	CT; intraoperative visualization	
Fujiyama et al. [15]	CTA/CTV; postoperative CTV confirming patency	
Kow et al. [16]	Catheter angiography confirmed shunt	
Guppy and Ochi [17]	CT/CTA	
Schlag et al. [18]	CCT/CTA; empty delta; postoperative CTA showed occlusion from entry to confluence	
Okada et al. [19]	Skull X-ray; CT; angiography	
Abdallah et al. [9]	CT; limited venography	
Arham and Zaragita [34]	CT/CTA/MRV	
Vakil and Singh [31]	CT/CTA; CTV/MRV; angiography (best practices)	
Thoeny et al. [28]	CT/CTA/CTV; MRA	
Nussbaum et al. [26]	CT/angiography	
Quiñones-Hinojosa et al. [29]	MRV to confirm thrombosis (following bullet extraction)	
Meyer et al. [37]	CTA and DSA in all patients	
Zafonte et al. (a) [24]	Angiography; CT	
Zafonte et al. (b) [24]		
Zafonte et al. (c) [24]		
Wang et al. [35]	CT/CTA/3D recon	
Suhendar et al. [33]	CT	
Zhu et al. [36]	CT; CTA/V; 3D CT; postop CT	
Mansour et al. [30]	CT; CTA; CTV (47 patients received CTV)	

### Management

Management was heterogenous (Table 3). Sinus repair/reconstruction was described in 10/24 cases (41.7%), decompression/craniectomy in 5/24 (20.8%), anticoagulation in 3/24 (12.5%), and ligation/segment isolation in 3/24 (12.5%).

**Table 3:** Management

Citation	Injury velocity	Intervention strategy	Reconstruction	Ligation	Decompression	Anticoagulation
Brune et al. [25]	High	Initial repair not watertight; patient seated day 9	No	No	No	No
Birk et al. [22]	High	Bilateral craniectomy (decompression) + ICP therapy	No	No	Yes	Not reported
Pricola et al. [20]	High	Occipital/suboccipital craniotomy, bullet & fragment removal, primary repair	Yes (primary repair)	No	No	Not reported
Haider et al. [21]	High	Occipital/suboccipital craniectomy; elevation of depressed fracture; mesh reconstruction	No (decompression)	No	Yes	No
Beaty et al. [23]	High	Suboccipital decompression; ligation of codominant TS; EVD—> VP shunt	No	Yes (TS)	Yes	No
Duke et al. [27]	Low	Supportive; no retrieval (patient critically ill neurologically)	NA	No	No	Not reported
Duda et al. [32]	High	Supportive; no endovascular extraction reported	NA	No	No	Not reported
Sani et al. [14]	Low	Temporalis fascia/muscle 'plug-and-patch' drawn in during extraction	Yes (plug-and-patch)	No	No	No
Fujiyama et al. [15]	Low	Bifrontal craniotomy; Gore-Tex patch + gelatin/fibrin; preserve antegrade flow	Yes (patch)	No	No	No
Kow et al. [16]	Low	Double concentric craniotomy; segment isolation/ligation anterior & posterior; shunt disconnection	No	Yes (segment isolation)	No	No
Guppy and Ochi [17]	Low	U-shaped craniectomy exposures; screws removed under direct vision; no antiplatelet	No (extraction + hemostasis)	No	No	No
Schlag et al. [18]	Low	Sutured closure; no interposition (segment already thrombosed)	Primary suture (segment thrombosed)	No	No	No
Okada et al. [19]	Low	Doughnut craniotomy; careful removal	No	No	No	No
Abdallah et al. [9]	High	PTFE interposition graft with proximal/distal clamps	Yes (PTFE graft)	No	No	LMWH x 48 h; aspirin × 3 months, beginning 2 days post-operatively
Arham and Zaragita [34]	Low	Hemostatic repair; small dural flaps; careful positioning; antibiotics	Small flap repair	No	No	Not specified
Vakil and Singh [31]	Mixed	Diagnostic recommendations	NA	NA	NA	NA
Thoeny et al. [28]	Low	Anesthesia-led multidisciplinary planning; blood products ready; CT reserved; Nail removed with vicegrip	NA	NA	NA	NA
Nussbaum et al. [26]	Low	Refinement of SSS repair technique; flow preservation	Yes	No	No	Not specified
Quiñones-Hinojosa et al. [29]	High	Conservative in case; literature includes urokinase thrombolysis	NA	NA	NA	No
Meyer et al. [37]	Mixed	Diagnostic comparison; DSA as gold standard	NA	NA	NA	NA
Zafonte et al. (a) [24]	High	Debridement; anticoagulation in 2; conservative in 1	NA	NA	Yes (one had elevation of depressed fracture)	Heparin- > warfarin in 2 cases, timing unspecified
Zafonte et al. (b) [24]	High					
Zafonte et al. (c) [24]	High					
Wang et al. [35]	Low	Extraction and repair; antibiotic prophylaxis; follow-up	Yes (local flap/repair)	No	No	No
Suhendar et al. [33]	Low	Craniotomy, extraction from transverse sinus	No	No	No	No
Zhu et al. [36]	Low	Bifrontal craniectomy; debridement; hematoma evacuation	Yes; muscle graft	Yes; SSS ligated	Yes; bifrontal craniectomy	No
Mansour et al. [30]	Mixed	Mostly observation; selective antithrombotics; rare endovascular therapy	None described	Not reported	Not systematically reported	Used in 2 venous sinus thrombosis cases, timing unknown; otherwise avoided

### Complications

Complications were explicitly absent in 13/24 cases (54.2%). Among reported adverse events, embolic complications were the most common (3/24, 12.5%), including pulmonary air embolism, pulmonary infarction from bullet embolism, and cardiac bullet embolism. Other reported complications were isolated or uncommon, including shunt dependence, postoperative filling defect/thrombosis, CSF leak/gram-negative meningitis, intraoperative brisk bleeding, and persistent neurologic sequelae (Table 4).

**Table 4:** Complications and outcomes

Citation	Complications	Outcome
Brune et al. [25]	Pulmonary air embolism (fatal)	Death
Birk et al. [22]	No major reported	Near-complete symptom resolution; improved Q-MRV flow
Pricola et al. [20]	Intraoperative brisk bleeding controlled	Excellent neuro outcome with restored proximal/distal flow
Haider et al. [21]	No major complications reported	Rapid improvement of papilledema/diplopia; good follow-up
Beaty et al. [23]	Shunt dependence	Favorable functional recovery
Duke et al. [27]	Pulmonary infarction	Not fully detailed; focus on embolism
Duda et al. [32]	Cardiac bullet embolism	Not detailed (patient nonsurvivable neurologically)
Sani et al. [14]	None reported	Excellent
Fujiyama et al. [15]	None reported	Good
Kow et al. [16]	None reported	Good
Guppy and Ochi [17]	Small filling defect on CTA	Good; returned to work
Schlag et al. [18]	None; occluded segment tolerated	Complete neurological recovery
Okada et al. [19]	None reported	Good
Abdallah et al. [9]	None reported (early)	Good; discharged day 5
Arham and Zaragita [34]	None	Good
Vakil and Singh [31]	Highlights venous thrombosis and infection risks	NA
Thoeny et al. [28]	Emphasis on catastrophic bleeding/air risk	Good; left homonymous hemianopsia (otherwise neurologically intact)
Nussbaum et al. [26]	None	Excellent; return to baseline reported
Quiñones-Hinojosa et al. [29]	NA	No focal neurologic deficits
Meyer et al. [37]	NA	CTA sensitivity lower; DSA often necessary
Zafonte et al. (a) [24]	Severe disability across series	Survival with severe disability
Zafonte et al. (b) [24]		
Zafonte et al. (c) [24]		
Wang et al. [35]	None reported	Good; complete recovery
Suhendar et al. [33]	None significant	Discharged on day 8 with GCS 15, no neurological deficits
Zhu et al. [36]	None significant	Good recovery; mild cognitive deficits; rehab; cranioplasty at 3 months
Mansour et al. [30]	High mortality	Vascular injury associated with worse discharge GOSE; SSS occlusion common

### Outcomes

Outcomes were generally favorable (Table 4). Good recovery or no focal neurologic deficit was reported in 18/24 cases (75.0%); 3/24 (12.5%) had persistent neurologic deficits at follow up, 1/24 (4.2%) died, and 2/24 (8.3%) lacked sufficient outcome detail. Survival was therefore documented or strongly implied in 21/24 cases (87.5%), with 1 explicit mortality and 2 cases with indeterminate survival/outcome detail.

## Discussion

This scoping review synthesizes published case reports and small case series describing penetrating injuries to the dural venous sinuses resulting from low-velocity penetrators, firearm-related trauma, and other penetrating mechanisms [17, 24, 26]. The included literature demonstrates that survival with meaningful recovery is achievable in patients who reach definitive neurosurgical care [17, 24, 26]. Across mechanisms and sinus segments, authors consistently emphasize meticulous surgical exposure, secure control of the involved sinus prior to foreign body manipulation, and tailored intraoperative decision-making regarding venous repair versus ligation [26–28]. Collectively, these reports illustrate an evolution in operative strategy, with contemporary management favoring deliberate visualization and reconstruction when feasible rather than blind packing or emergent ligation [29–31].

### Mechanism-specific injury patterns and outcomes

Low-velocity penetrating mechanisms, including nail guns, drywall screws, and captive-bolt injuries, were consistently described as producing focal sinus lacerations with limited surrounding parenchymal injury, thereby facilitating controlled operative exposure [15, 19, 25]. Under these conditions, circumferential sinus exposure was feasible, allowing removal of the penetrating object under direct visualization followed by hemostasis achieved via localized repair strategies [22, 32]. Several reports documented favorable neurological outcomes following such approaches, including return to baseline function or minimal residual deficits [22, 23]. The consistency of these outcomes across independent case reports supports the conclusion that low-velocity sinus injuries are particularly amenable to flow-preserving surgical management when appropriately exposed [15, 19, 32].

By contrast, firearm-related penetrating injuries were substantially more heterogeneous, frequently characterized by comminuted skull fractures, retained ballistic fragments, and involvement of posterior sinus segments or the torcular confluence, often in conjunction with significant parenchymal damage [9, 31]. Despite this complexity, several cases demonstrated that aggressive yet controlled surgical intervention can yield favorable outcomes in selected patients. Successful decompression, removal of ballistic material, and direct dural sinus repair were reported even in anatomically critical regions such as the torcula [29, 30]. These cases emphasize that firearm-related sinus injuries should not be considered universally nonsurvivable when venous control and reconstruction are technically achievable [30, 31].

### Segment-specific surgical decision-making

Decision-making regarding dural venous sinus ligation versus repair varied according to sinus segment and intraoperative findings. Several reports described injuries involving the anterior portion of the superior sagittal sinus that were managed with ligation or partial occlusion without catastrophic neurological consequences, particularly when performed under controlled conditions with adequate exposure [18, 34]. However, other cases reported venous congestion or infarction following compromise of the superior sagittal sinus, highlighting interpatient variability in venous tolerance [26]. These findings suggest that anterior sinus ligation may be acceptable in selected circumstances but should not be considered universally benign [26, 34].

Injuries involving the posterior superior sagittal sinus, torcular confluence, or transverse sinus were more consistently associated with adverse neurological sequelae when venous outflow was compromised. Multiple reports documented intracranial hypertension, venous infarction, or neurological deterioration in the setting of posterior sinus occlusion [35, 36]. Consequently, authors frequently favored flow-preserving strategies in these regions, including decompression of extrinsic compression or direct reconstruction, with improved postoperative outcomes reported when venous patency was restored [16, 29]. These observations reinforce the importance of segment-specific risk stratification when managing penetrating sinus injuries.

### Surgical technique: exposure, control, and reconstruction

Across all mechanisms, wide exposure allowing circumferential visualization of the involved sinus emerged as the most consistent determinant of successful surgical management. Authors repeatedly described the use of wide craniotomy or concentric donut craniotomy techniques to stabilize penetrating objects and permit proximal and distal sinus control prior to extraction [26–28]. This approach reduces the risk of uncontrolled hemorrhage and enables deliberate inspection of sinus leaflets and intraluminal pathology.

Reconstruction strategies varied according to defect size, tissue integrity, and the specific sinus segment involved. Small sinus defects were frequently managed with direct suturing or autologous tissue patches, whereas larger or segmental injuries required patch angioplasty or interposition grafting [20, 30]. Several reports documented successful reconstruction using synthetic materials, demonstrating that durable venous repair is feasible even in challenging or resource-limited settings [31]. Hemostatic adjuncts, including gelatin-thrombin matrix and oxidized cellulose, were commonly employed to control venous bleeding during repair and to facilitate definitive reconstruction [14, 21].

### Imaging: diagnostic and surveillance imperatives

Venous-phase imaging played a critical role in both initial diagnostic evaluation and postoperative surveillance. CT venography and MR venography were used to assess sinus patency, identify thrombosis, and evaluate extrinsic compression from fracture fragments or hematoma [24, 37]. Several authors noted that radiographic sinus narrowing may reflect either compression or thrombosis, a distinction that directly influences decisions regarding surgical decompression versus conservative management [35].

Delayed venous imaging was particularly important, as multiple reports documented the development of dural venous sinus thrombosis on follow-up studies despite initially reassuring postoperative imaging [33, 36]. These observations support routine serial venous imaging in patients with penetrating sinus injury, especially when posterior sinus segments are involved, given the higher risk of clinically significant venous outflow obstruction [22].

### Catastrophic complications: air embolism and delayed thrombosis

Venous air embolism emerged as a rare but catastrophic complication within the included literature. Fatal outcomes were reported in cases where sinus lacerations were incompletely sealed and patients were mobilized prematurely, permitting ingress of air into the venous system [9, 17]. Authors emphasized the importance of achieving watertight sinus closure and maintaining strict positioning precautions until venous integrity is assured [28, 34]. These reports highlight the unique vulnerability of non-collapsible dural venous sinuses to air entry during the perioperative and early postoperative periods.

Delayed dural venous sinus thrombosis was more frequently reported and occurred across mechanisms and sinus segments. Thrombosis was attributed to direct sinus injury, altered venous flow following repair or compression, and postoperative changes in hemodynamics or coagulation [31, 35, 36]. The recurrence of this complication across independent reports underscores the importance of structured surveillance and individualized management strategies, including consideration of anticoagulation when the hemorrhagic risk is acceptable.

### Anticoagulation in traumatic DVST: navigating hemorrhagic risk

Anticoagulation practices varied considerably among the included studies, reflecting the tension between preventing thrombotic propagation and exacerbating intracranial hemorrhage. Some authors initiated anticoagulation after stabilization of intracranial bleeding, whereas others favored conservative management in light of perceived hemorrhagic risk [14, 21, 32]. Favorable outcomes were reported in selected anticoagulated patients, although authors consistently emphasized individualized decision-making rather than rigid protocolized treatment [26, 33].

This heterogeneity highlights persistent uncertainty regarding optimal timing, choice, and intensity of anticoagulation in traumatic DVST following penetrating sinus injury. The available evidence remains insufficient to support standardized regimens, and future studies will be required to clarify risk–benefit profiles in distinct mechanistic and anatomical contexts.

### Limitations

There are several key limitations to this study. First, this review is limited by the quality and design of the underlying literature, which consists almost entirely of case reports and small retrospective studies. As such, completion of a formal meta-analysis is not possible, as is the case with estimation of effect sizes and other formal comparative measures. The resulting narrative synthesis inherently introduces interpretive subjectivity, and as such, recommendations should be regarded as hypothesis-generating rather than declarative. Restriction to English-language and indexed publications also raises the possibility of language and database bias. Second, as outlined above, penetrating injury to the dural venous sinuses is inherently heterogeneous. Clinical variability may limit generalizability, as included patients span low-velocity nail-gun and drywall-screw injuries to high-energy firearm and shrapnel injury. There is further diversity in sinus segment involvement, associated parenchymal damage, presentation timing, and hemodynamic status. Furthermore, operative techniques, imaging protocols, anticoagulation strategies, and outcome definitions differ across institutions and time periods. This clinical heterogeneity prevents standardized pooling or subgroup analysis by mechanism, segment, or repair approach. Additionally, incomplete reporting of patient-specific collateral anatomy, intraoperative decision-making, and longitudinal follow-up impairs assessment of *i*) the effects of anatomic variations on clinical managements, and *ii*) late complications including chronic DVST and venous hypertension. Lastly, the literature lacks studies comparing venous sinus repair versus ligation, specific reconstruction materials, or anticoagulation regiments in the setting of penetrating sinus injury. As such, any proposed management framework rests on observational evidence, expert opinion, or extrapolation from non-penetrating dural venous sinus injury literature. Furthermore, publication bias is likely to favor technically successful reconstruction and unusual complications, overestimating the frequency of both favorable outcomes and rare events including post-operative venous air embolism.

## Conclusion

Penetrating injuries to the dural venous sinuses demand a segment-specific management approach incorporating early venous imaging, meticulous wide exposure with proximal and distal sinus control, and deliberate choice between repair and ligation. Modern microsurgical techniques, synthetic grafts, and hemostatic adjuncts have made flow preserving reconstruction increasingly feasible, even at junctional regions including the torcular confluence. Low-velocity penetrators managed with controlled exposure and repair achieve excellent outcomes, while firearm injuries demonstrate greater variability requiring careful surveillance for air embolism and delayed sinus thrombosis. Vigilant monitoring for post-traumatic DVST and prevention of air embolism through patient positioning remain essential to optimize survival and neurological recovery in this rare but catastrophic injury pattern.

## Supplementary Information

Below is the link to the electronic supplementary material.

## Data Availability

No datasets were generated or analysed during the current study.
