# Supplementary material for: Penetrating dural venous sinus injury: a scoping review of clinical presentation, operative management, and prognosis
Source: Neurosurg Rev. 2026 Sep 3;49(1):548. doi: 10.1007/s10143-026-04451-2 (PMC13538148; doi:10.1007/s10143-026-04451-2)
Supplement: Supplementary file 1 [file 10143_2026_4451_MOESM1_ESM.docx]

Supplemental table 1

| **Database** | Full Search Syntax | Number of Search Results |
| --- | --- | --- |
| PubMed | ("dural venous sinus" OR "superior sagittal sinus" OR "transverse sinus") AND ("injury" OR "laceration" OR "thrombosis") AND ("gunshot" OR "ballistic" OR "penetrating") | 46 |
| Embase | ('dural venous sinus injury'/exp OR 'venous sinus'/exp OR 'superior sagittal sinus':ab,ti  OR 'transverse sinus':ab,ti OR torcula:ab,ti) AND ('gunshot wound'/exp OR 'gunshot':ab,ti OR 'ballistic injury'/exp OR 'missile injury':ab,ti OR 'penetrating brain injury':ab,ti OR 'cranial gunshot':ab,ti) AND [humans]/lim AND [english]/lim | 25 |
| Scopus | dural venous sinus or venous sinus or cerebral venous sinus or superior sagittal sinus or transverse sinus or torcula AND gunshot or gunshot wound or ballistic or missile injury or penetrating brain injury or cranial gunshot | 2 |
| PubMed Central | (("dural venous sinus"[Title/Abstract] OR "venous sinus"[Title/Abstract] OR "cerebral venous sinus"[Title/Abstract] OR "superior sagittal sinus"[Title/Abstract] OR "transverse sinus"[Title/Abstract] OR "torcula"[Title/Abstract])) AND (("gunshot"[Title/Abstract] OR "gunshot wound"[MeSH Terms] OR "ballistic"[Title/Abstract] OR "missile injury"[Title/Abstract] OR "penetrating brain injury"[Title/Abstract] OR "cranial gunshot"[Title/Abstract])) | 2 |
